# A Positive Role of Cadherin in Wnt/β-Catenin Signalling during Epithelial-Mesenchymal Transition

**DOI:** 10.1371/journal.pone.0023899

**Published:** 2011-08-31

**Authors:** Sara Howard, Tom Deroo, Yasuyuki Fujita, Nobue Itasaki

**Affiliations:** 1 MRC National Institute for Medical Research, London, United Kingdom; 2 MRC Laboratory for Molecular Cell Biology and Cell Biology Unit, Department of Cell and Developmental Biology, University College London, London, United Kingdom; 3 Division of Molecular Oncology, Institute for Genetic Medicine, Hokkaido University, Sapporo, Hokkaido, Japan; Institute of Science and Technology Austria, Austria

## Abstract

The Wnt/β-catenin signalling pathway shares a key component, β-catenin, with the cadherin-based adhesion system. The signalling function of β-catenin is conferred by a soluble cytoplasmic pool that is unstable in the absence of a Wnt signal, whilst the adhesion function is based on a cadherin-bound, stable pool at the membrane. The cadherin complex is dynamic, allowing for cell-cell rearrangements such as epithelial-mesenchymal transition (EMT), where the complex turns over through internalisation. Potential interplay between the two pools remains poorly understood, but cadherins are generally considered negative regulators of Wnt signalling because they sequester cytoplasmic β-catenin. Here we explore how cellular changes at EMT affect the signalling capacity of β-catenin using two models of EMT: hepatocyte growth factor (HGF) treatment of MDCK cells, and gastrulation in embryonic development. We show that EMT not only provides a pool of signalling-competent β-catenin following internalisation of cadherin, but also significantly facilitates activation of the Wnt pathway in response to both Wnt signals and exogenous β-catenin. We further demonstrate that availability of β-catenin in the cytoplasm does not necessarily correlate with Wnt/β-catenin pathway activity, since blocking endocytosis or depleting endogenous cadherin abolishes pathway activation despite the presence of β-catenin in the cytoplasm. Lastly we present data suggesting that cadherins are required for augmented activation of the Wnt/β-catenin pathway *in vivo*. This suggests that cadherins play a crucial role in β-catenin-dependent transcription.

## Introduction

During embryogenesis and in adulthood, cells undergo dynamic morphological rearrangements, including changes in cell-cell adhesion, which are essential for driving both morphogenetic processes, such as gastrulation, and differentiation into distinct tissues. These cellular changes critically depend upon precise interactions between neighbouring cells and tight regulation of gene expression; dysregulation of either may lead to perturbed cell proliferation, adhesion, migration or differentiation, all of which are hallmarks of diseases such as cancer.

The cadherin/catenin-based adhesion system is the major means by which cells adhere to one another. β-catenin, a central structural component of this adhesion complex, also acts as a transcriptional co-activator in the Wnt signalling pathway, a pathway used reiteratively during development to control cell fate decisions and one implicated in cancer in many tissues. The current, and somewhat uncontested, understanding of this pathway is that Wnt receptor activation inhibits the degradation of newly synthesised β-catenin, allowing for a *de novo* synthesised, cadherin-free form of β-catenin to enter the nucleus and, together with LEF/TCF, activate Wnt target genes [1]. In fact, it has been shown that Wnt signalling generates a form of β-catenin that binds TCF but not cadherin, suggesting that β-catenin used for transcription is molecularly distinct from that used for adhesion [2]. Despite this finding, there has been much speculation over whether the cadherin-bound and signal transduction pools of β-catenin are functionally interchangeable [3], [4], [5], [6], [7]. If so, this might allow for a Wnt ligand-independent induction of Wnt target genes, whereby β-catenin released from the membrane is used directly for signalling. In support of this idea, it has recently been shown in a cancer cell line that dissociation of adherens junctions by lysophosphatidic acid results in a release of β-catenin, which, in a mutated stabilised form, translocates into the nucleus [8]. However, it is not known whether this occurs with native β-catenin. This utilisation of cadherin-bound β-catenin in Wnt signalling is interesting, as cadherins are generally considered negative regulators of the pathway, through their sequestration of β-catenin, and it has been shown that increased and decreased levels of cadherin expression inhibit and activate β-catenin-dependent transcription, respectively [9], [10], [11], [12], [13], [14], [15].

The concept of ligand-independent Wnt signalling is further suggested by the fact that in some developmental situations there appears to be no obligate correlation between Wnt ligand expression and Wnt pathway activation. This is the case for migrating neural crest cells; despite not expressing any known Wnt ligand, the Wnt pathway is active in these cells [16] and is required for them to form cranial ganglia [17]. Another example is gastrulation, where epiblast cells delaminate and migrate laterally to form paraxial mesoderm. Wnt/β-catenin activity is required in these migrating cells in order for them to contribute to the mesoderm; in mouse embryos harbouring mutations in either *Wnt3a* or the Wnt-regulated mesodermal transcription factor *Tbx6*, these delaminated cells form additional neural tubes instead of somites [18], [19]. This Wnt activity requirement occurs in the absence of any significant upregulation of Wnt ligand expression.

Importantly, both of these events see cells undergoing epithelial-mesenchymal transition (EMT), a process involving dissociation of adherens junctions and changes in cell morphology. The above *in vivo* events could, therefore, be explained by an alternative phenomenon: differential sensitivity to Wnt signals between epithelial and mesenchymal cells. Compared to epithelial cells, mesenchymal cells generally exhibit a much less regimented structure and weaker intercellular adhesion. Thus, it is possible that the cytoarchitectural changes that occur at EMT render the mesenchymally transformed cells more sensitive to Wnt signals originating from neighbouring tissue, the Wnt ligand-expressing ectoderm in the cases of migrating neural crest and mesodermal cells.

To gain more insight into the relationship between the two functions of β-catenin, and to explore how cellular changes at EMT affect the signalling capacity of β-catenin we used two models of EMT: hepatocyte growth factor (HGF) treatment of MDCK cells, and gastrulation in embryonic development. We find that cadherins do not merely provide a pool of β-catenin capable of feeding into the Wnt pathway, but rather play a positive role in rendering β-catenin competent in signalling.

## Results

### Mesenchymal Cells Show an Enhanced Wnt Transcriptional Response Compared to Epithelial Cells

**Figure 1:**
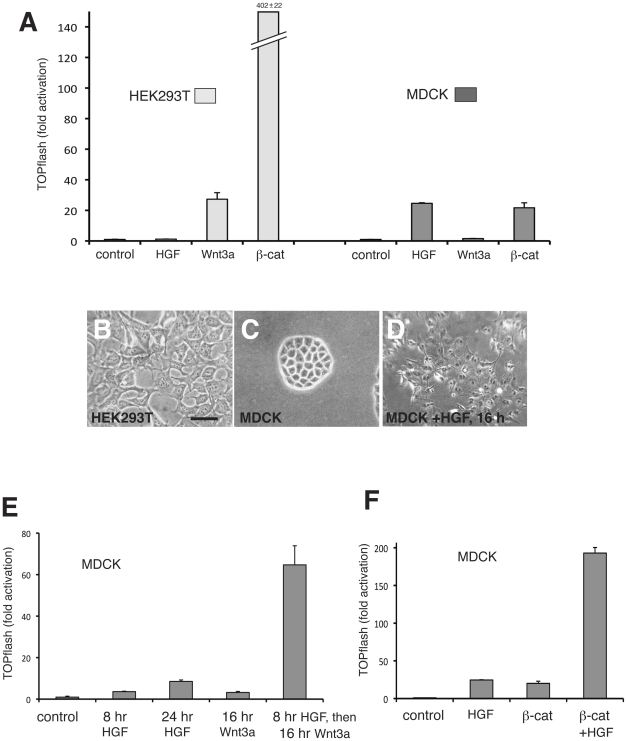
Mesenchymal cells show strong Wnt transcriptional response compared to epithelial cells. (A) TOPflash reporter assay in HEK293 and MDCK cells treated with 250 ng/ml HGF or Wnt3a conditioned medium, or transfected with β-catenin. HGF causes mild activation of TOPflash in MDCK cells. HEK293 cells exhibit an enhanced Wnt transcriptional readout relative to MDCK cells. (B–D) Appearance of HEK293 cells (B), MDCK cells (C) and MDCK cells treated with HGF for 16 hours (D). MDCK cells scatter and adopt a mesenchymal morphology akin to HEK293 cells following HGF treatment. Scale bar; 50 µm. (E) TOPflash reporter assay in MDCK cells treated with HGF or Wnt3a alone, or pre-treated with HGF for 8 hours before exposure to Wnt3a. HGF treatment augments the transcriptional response to Wnt3a. (F) TOPflash reporter assay in MDCK cells treated with HGF or transfected with β-catenin alone, or transfected with β-catenin and treated with HGF for the final 16 hours before harvesting. HGF treatment augments the transcriptional response to transfected β-catenin.

For studying the Wnt signalling pathway, HEK293 cells are widely used as they are very responsive to exogenous Wnt ligands and transfected β-catenin, as judged by activation of the established β-catenin/LEF/TCF reporter TOPflash (Fig. 1A). In contrast, MDCK cells hardly respond to Wnt3a medium, and show only a mild activation by β-catenin transfection (Fig. 1A). HEK293 cells and MDCK cells are morphologically distinct. Although epithelial in origin, HEK293 cells grow in a flattened and scattered manner (Fig. 1B), while MDCK cells maintain typical epithelial features such as apical-basal polarity, tight junctions and adherens junctions [20] and display a cobblestone-like appearance (Fig. 1C). This tightly compacted epithelial morphology of MDCK cells can be converted to a mesenchymal one by treatment with HGF, which causes a breakdown of intercellular junctions and an acquisition of cell motility, processes typical of EMT [21], [22] (Fig. 1D). It has previously been reported that HGF treatment causes mild activation of TOPflash in MDCK cells [23], which we confirmed (Fig. 1E,F). Notably, when MDCK cells are transformed to a mesenchymal morphology by HGF before being treated with Wnt3a, the Wnt pathway readout is significantly enhanced (Fig. 1E). This is also the case for β-catenin transfection; HGF treatment significantly augments the effect of exogenous β-catenin (Fig. 1F). These results suggest that fundamental changes that occur when a cell switches from an epithelial to a mesenchymal morphology facilitate the activation of the Wnt pathway, not only in response to Wnt signals but also to exogenous β-catenin.

### HGF-induced EMT of MDCK Cells Results in Redistribution of β-catenin from the Membrane to the Cytoplasm and Causes Activation of Wnt-responsive Genes

**Figure 2:**
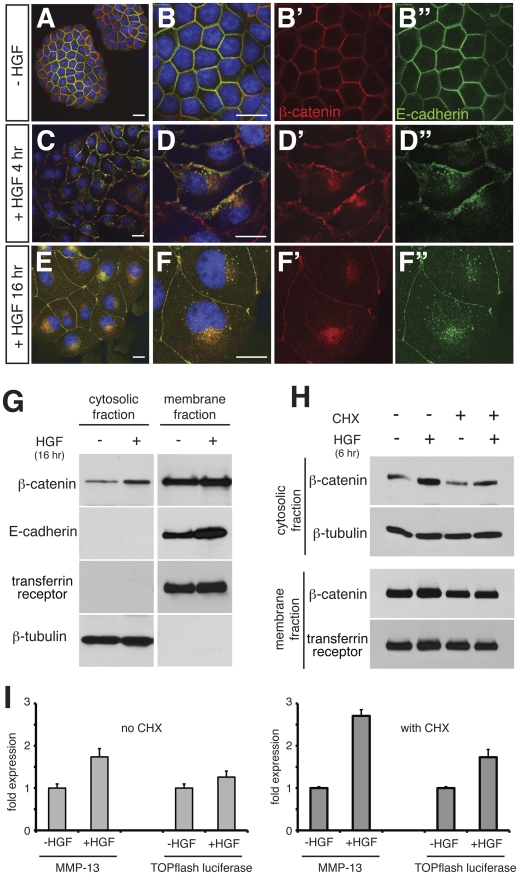
HGF-induced EMT of MDCK cells induce redistribution of β-catenin and causes activation of Wnt-responsive genes. (A–F) Confocal microscopy of MDCK cells showing β-catenin (red), E-cadherin (green) and nucleus (blue) after HGF treatment as indicated. Without HGF (A,B), β-catenin is mostly co-localised with E-cadherin at the cell membrane. HGF treatment causes flattening and spreading of cells (C–F), along with internalisation of β-catenin and E-cadherin. Although both E-cadherin and β-catenin are mainly localised in a perinuclear region, they are not strictly colocalised, as internalised E-cadherin is seen in a punctate manner while β-catenin is distributed relatively broadly. Scale bars, 20 µm. (G) Western blot analyses of cytosolic and membrane fractions of MDCK cells after treatment with HGF. β-catenin is present in the cytosolic fraction at a low level in untreated MDCK cells, and is increased by HGF treatment. E-cadherin is detectable only in the membrane fraction, as is transferrin receptor (loading control). The amount of E-cadherin increases consistently in the membrane fraction following HGF treatment. β-tubulin serves as a loading control of the cytosolic fraction. (H) Western blot analyses of MDCK cells showing that cytosolic β-catenin increases in response to HGF both in the absence and presence of cycloheximide (CHX). (I) The relative levels of Wnt target genes (transfected TOPflash reporter and endogenous target MMP-13) are increased by 4 hours of HGF treatment both in the absence and presence of cycloheximide (CHX) as shown by quantitative PCR. In the condition with no CHX, *P*-values were *P*<0.001 for MMP13 and *P* = 0.046 for TOPflash luciferase. In the condition with CHX, *P*<1×10^−5^ for MMP-13, *P*<0.002 for TOPflash luciferase.

**Figure 3:**
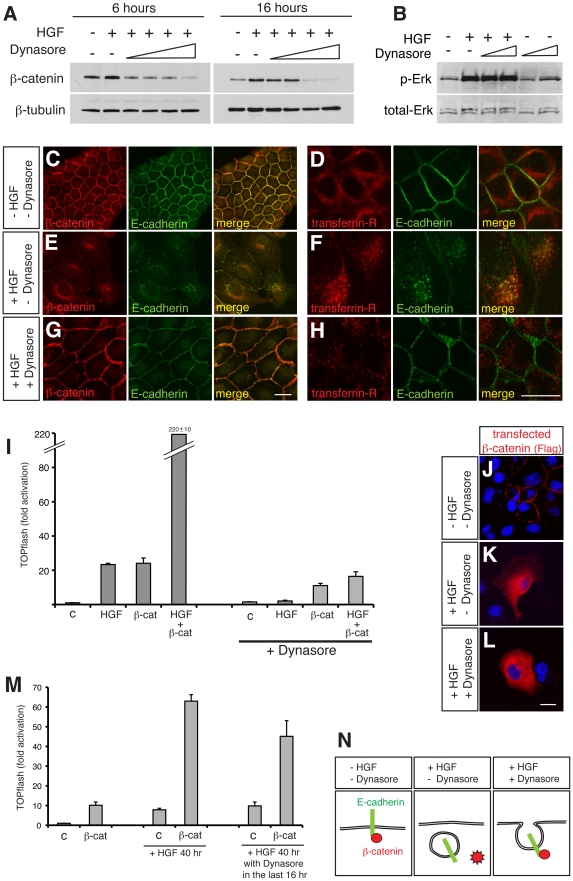
Stimulation of β-catenin signals by HGF is dependent on endocytosis. (A) Western blot analyses of the cytosolic fraction of MDCK cells after treatment with HGF and increasing amounts of Dynasore (50, 100, 200 and 400 µM) for 6 and 16 hours shows a dose-dependent reduction in cytosolic β-catenin accumulation by HGF. (B) Western blot analysis of cytosolic fraction of MDCK cells after treatment with HGF and Dynasore (100 and 400 µM) for 30 minutes. Blocking endocytosis by Dynasore does not interfere with HGF signalling-induced phsophorylation of Erk (p-Erk). (C–H) Confocal microscopy of MDCK cells showing E-cadherin (green) and either β-catenin (red in C,E,G) or transferrin receptor (red in D,F,H) after treatment with HGF without or with Dynasore (400 µM) for 16 hours. In the absence of HGF or Dynasore (C,D), β-catenin and E-cadherin are localised at the cell membrane (C, also shown in Fig. 2A,B) while transferrin receptor is mostly in the subcortical area (D). HGF treatment (E,F) causes internalisation of β-catenin and E-cadherin (E, also shown in Fig. 2E,F) and movement of transferrin receptor to a perinuclear location, where it colocalises with internalised E-cadherin (F). In the presence of Dynasore, the ability of HGF to induce internalisation of β-catenin and E-cadherin is abolished, and they both remain at the cell membrane (G). Scale bars; 20 µm. (I) TOPflash reporter assay in MDCK cells with control (c) or β-catenin (β-cat) transfection and HGF treatment (similar to Fig. 1F), in the absence or presence of Dynasore (400 µM). There is a reduction in Wnt pathway readout by both HGF and HGF plus β-catenin in the presence of Dynasore. HGF and Dynasore were added to transfected cells for the final 16 hours of incubation. Data were all normalised to the control without Dynasore. (J–L) Confocal microscopy of MDCK cells transfected with Flag-tagged β-catenin (red), treated with HGF and Dynasore as indicated, counterstained with DAPI (blue). Exogenous β-catenin is localised at the cell membrane in the absence of HGF (J), whereas HGF-treated cells show exogenous β-catenin in the cytoplasm, both in the absence (K) and presence (L) of Dynasore. Scale bar; 20 µm. (M) TOPflash reporter assay in MDCK cells with β-catenin transfection and HGF and Dynasore (200 µM) treatment. Where applicable, HGF were first added to cells for 24 hours to make cells mesenchymal, before adding Dynasore for 16 hours (total 40 hours of HGF). Dynasore attenuates the effect of transfected β-catenin in the mesenchymally transformed cells. *P = 0.011. (N) Schematic diagram summarising the requirement for the release of an active form of β-catenin into the cytosol.

In MDCK cells, β-catenin is predominantly at the membrane, where it colocalises with E-cadherin (Fig. 2A,B). Treating cells with HGF causes cells to flatten and scatter, and induces redistribution of both β-catenin and E-cadherin to the intracellular space, which commences within 4 hours (Fig. 2C,D) and is fully attained by 16 hours (Fig. 2E,F). This process is likely mediated by activation of Src tyrosine kinase and the subsequent phosphorylation of E-cadherin, which leads to dissociation of catenins from the cadherin complex and breakdown of the adherens junction [24]. Immunocytochemical analyses revealed that E-cadherin is largely distributed in a punctate manner in HGF-treated MDCK cells (Fig. 2C–F), which is in agreement with previous studies showing that internalised E-cadherin is, upon src-mediated EMT, located in the endosome and subsequently in the lysosome for degradation or recycling [25] (see also Fig. 3F). We found that β-catenin tends to accumulate at a perinuclear location, as reported in other systems [26], although not in a punctate manner (Fig. 2D′,2F′) and hence not exactly colocalising with E-cadherin in the cytoplasm. Cell fractionation analyses show that E-cadherin is always detected in the insoluble fraction (membrane fraction including endosomal vesicles) and not in the cytosolic fraction even after HGF treatment, while β-catenin in the cytosolic fraction increases in response to HGF treatment at 6 and 16 hours (Fig. 2G,H). Thus, it appears that once E-cadherin is internalised by HGF stimulation, β-catenin is dissociated from the cadherin complex and becomes abundant in the cytosol, while E-cadherin remains in membrane vesicles. The increase of β-catenin in the cytosolic fraction by HGF is seen both in the absence and presence of cycloheximide at the 6 hour time point (Fig. 2H), suggesting that, at least within the initial 6 hours, this increase is not due to *de novo* β-catenin synthesis, but rather to a possible movement of pre-existing β-catenin from the membrane to the cytosol. We did not detect a reduction in β-catenin in membrane fraction after HGF treatment, presumably due to the relatively large amount in the membrane fraction.

To test whether the HGF-induced EMT causes activation of Wnt target genes, we looked at the expression levels of both the potential endogenous target MMP-13 [27], [28] and the transfected TOPflash reporter in MDCK cells treated with cycloheximide for 6 hours and/or HGF using quantitative RT-PCR, and found that HGF increases the transcription of these genes even in the presence of cycloheximide (Fig. 2I). The increase in expression of these transcripts by HGF was slightly higher in the presence of cycloheximide than in the absence of cycloheximide, possibly due to a block of negative feedback mechanisms that are often in operation in Wnt/β-catenin signalling. These results agree with the idea that β-catenin in the membrane pool is likely to contribute to the transcriptional activation of Wnt target genes upon HGF treatment.

### Endocytosis is Required for HGF's Stimulation of Canonical β-catenin Signals

To verify the shift of β-catenin from the membrane to the cytoplasm, we examined the role of endocytosis. E-cadherin undergoes a dynamic turnover; it is continuously internalised in a Dynamin- and Rab5-dependent manner, then is either recycled to the cell surface or degraded [29], [30], [31], [32]. Following activation of tyrosine kinases such as Src and the HGF receptor c-Met, the cytoplasmic tail of E-cadherin is phosphorylated, which directs internalised E-cadherin to lysosomal degradation [21], [25]. Concurrently, β-catenin is also phosphorylated at specific tyrosine residues, particularly at Y142 and Y654, which reduces its affinity for α-catenin and E-cadherin, respectively [33], [34], [35], resulting in breakdown of the cadherin complex intracellularly. To examine the requirement of endocytosis, we used dynasore, a potent inhibitor of dynamin-dependent endocytosis [36]. Dynasore treatment alone does not affect the amount of cytosolic β-catenin in MDCK cells after 16 hours (data not shown), but does block the HGF-dependent increase of β-catenin in the cytosol, both at 6 and 16 hours (Fig. 3A). Dynasore does not affect HGF signalling per se, as revealed by Erk phosphorylation (Fig. 3B), or general transcriptional activity, as measured by Renilla luciferase in the control of the reporter assay (data not shown). Immunocytochemical analyses showed that upon HGF treatment for 6 or 16 hours, E-cadherin partially colocalises with endocytosed transferrin receptor (Fig. 3F and data not shown). In the presence of dynasore, however, internalisation of E-cadherin is blocked and both β-catenin and E-cadherin remain at the cell membrane (Fig. 3G,H and data not shown). Under these conditions, Wnt pathway readout by both HGF and HGF in combination with β-catenin is significantly reduced (Fig. 3I), suggesting requirement of endocytosis for β-catenin to activate transcription in response to HGF. It is noted that the susceptibility of overexpressed β-catenin to endocytosis inhibition is observed not only in the condition where HGF and Dynasore are applied simultaneously (Fig. 3I), but also when cells are pre-treated with HGF for 24 hours (and are hence transformed to a mesenchymal morphology) before the addition of Dynasore (Fig. 3M). This suggests that endocytosis is required not merely for HGF to carry out an immediate effect, but also for augmented activation of the Wnt/β-catenin pathway in pre-transformed cells.

One possible mechanism for the requirement of endocytosis is the release of β-catenin from E-cadherin following internalisation of the complex, where HGF stimulates the mobilisation of β-catenin from the membrane. It is worth noting that following transfection of *β-catenin* DNA, exogenous β-catenin is found in the cytoplasm in cells treated with HGF, regardless of absence or presence of dynasore (Fig. 3K,L). Nevertheless, dynasore-treated cells show only weak transcriptional activation after β-catenin transfection (Fig. 3I), suggesting that a large proportion of overexpressed β-catenin is susceptible to the inhibition of endocytosis. Thus, it seems that it is not stability or availability in the cytoplasm, but rather the process of localising to the membrane and then being endocytosed, that is crucial for the transcriptional activity of β-catenin. This is supported by the finding that there is no significant difference in the TOPflash readout in MDCK cells transfected with full length β-catenin and those with a constitutively-active non-degradable form of β-catenin, at least when cells have an epithelial morphology or are lacking E-cadherin (see Fig. 5G). Another possible mechanism is the involvement of factors sensitive to endocytosis inhibition that are required for the transcriptional activity of β-catenin. In either case, our results underscore the importance of cellular endocytosis processes in transcriptional activation by β-catenin and show that the availability of β-catenin in the cytosol does not necessarily reflect its transcriptional activity.

### Ability to bind cadherin is required for β-catenin's transcriptional activity

We next wanted to test whether the augmented activation of β-catenin-dependent signalling by endocytic processes requires the endocytosis of the E-cadherin/β-catenin complex itself. For this purpose we used various mutant forms of β-catenin that show different affinities for its binding partners of the adhesion complex. The cadherin-catenin complex is a substrate for tyrosine kinases, suggesting phosphorylation as a potential mechanism for regulating the state of the complex [33]. It has been shown that these phosphorylation events are primarily involved in the regulation of adherens junction integrity and cell-cell adhesion [34], [35], [37], [38], [39], and that phosphomimetic and phospho-resistant forms of β-catenin affect its transcriptional activity in various cell types [34], [38], [39]. For example, the Y654E mutation reduces the affinity of β-catenin to cadherins [35], whereas Y142E β-catenin has a reduced affinity for α-catenin [38]. Although both of the forms are defective in forming the cadherin-catenin-cytoskeleton complex, the former does not bind to cadherin while the latter does.

**Figure 4:**
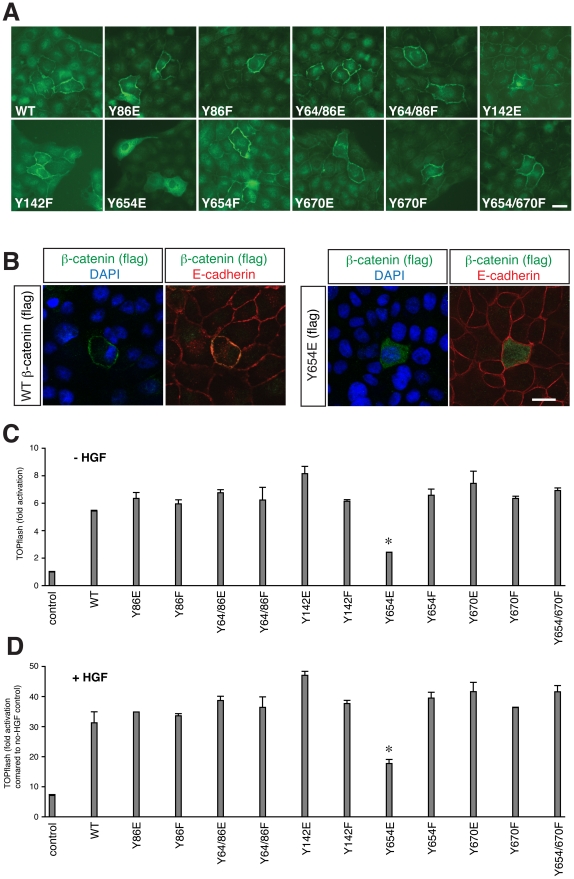
A form of β-catenin that does not localise to the cell membrane shows low transcriptional activity. (A) Fluorescent microscopy of MDCK cells immunostained for transfected Flag-tagged β-catenin of wild type (WT) and various mutant forms as indicated. Exogenous β-catenin, detected by anti-Flag antibody, is localised to the cell membrane, except for the Y654E mutant, which shows cytoplasmic distribution. Scale bar, 20 µm. (B) Similar analyses as (A), with co-staining with E-cadherin (red) and nuclei (blue). Wild type β-catenin co-localises with E-cadherin, whereas Y654E does not. Scale bar, 20 µm. (C, D) TOPflash reporter assay of the β-catenin mutants in MDCK cells, without (C) or with (D) HGF in the medium. The fold activation was normalised against cells transfected with control DNA without HGF treatment. Transcriptional activation is similar amongst all the constructs with the exception of Y654E, which gives a much-reduced transcriptional readout (*P<2×10^−7^ compared to other β-catenin constructs) in both conditions.

When transfected into MDCK cells, exogenous wild type β-catenin localises predominantly at the membrane, where it colocalises with E-cadherin (Fig. 4A,B; see also Fig. 3J). Among 11 mutant forms of β-catenin, only Y654E does not localise at the membrane, but is instead widely expressed throughout the cytoplasm (Fig. 4A,B). This is in agreement with the reduced affinity of Y654E to cadherins [35]. Remarkably, only this mutant is less able to induce TOPflash expression compared with wild type and other mutated forms of β-catenin (Fig. 4C), suggesting that the ability of β-catenin to bind to cadherins is crucial for its transcriptional activity. Moreover, addition of HGF to the medium sustains the relative trend of TOPflash activities in all tested constructs including the low activity of Y654E, despite augmented activities as a whole (Fig. 4D). Hence it appears that the crucial event in the augmented transcriptional activity of β-catenin during EMT is the process of β-catenin binding to cadherin and subsequently detaching from it, the latter of which is initiated by endocytosis. This also implies that β-catenin derived from the cadherin complex may be distinct from a newly translated β-catenin that has never bound to cadherin; the former can provide transcriptional activation of the Wnt pathway while the latter cannot. It is as if β-catenin is somehow primed for transcriptional activity by undergoing interaction with and detachment from cadherin. The priming effect is yet to be clarified, but at least it does not appear to be phosphorylation of Y654. The result also confirms that the mere availability in the cytoplasm is not sufficient in MDCK cells.

### Cadherin is required for the increase in cytoplasmic β-catenin and for transcriptional activation in response to Wnt and β-catenin

**Figure 5:**
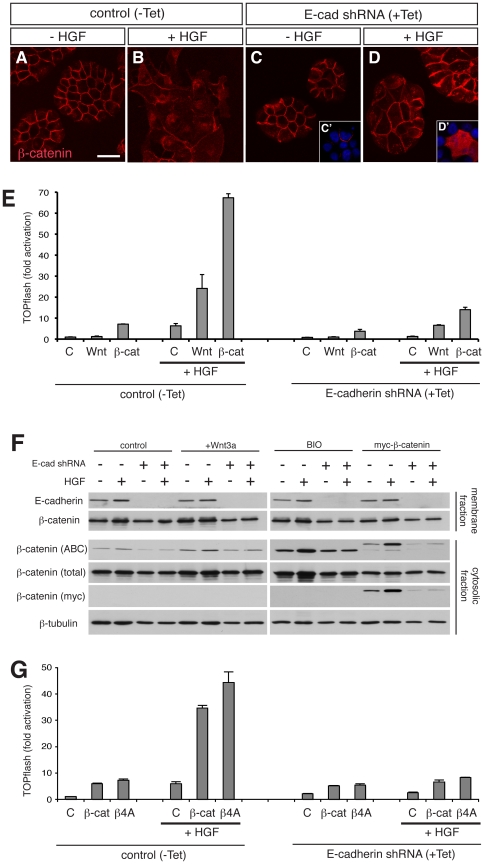
Cadherins are required for transcriptional activation by Wnt3a and β-catenin. (A–D) Confocal microscopy of MDCK cells without (control, −Tet) or with (+Tet) induction of E-cadherin shRNA expression by tetracycline, after treatment without or with HGF, stained for β-catenin. Insets in (C) and (D) (C′,D′) are exogenous β-catenin detected by myc tag. For the exogenous β-catenin in the presence of E-cadherin, see Fig. 3J,K. E-cadherin-depleted cells do not scatter by HGF and the majority of β-catenin remains on the cell membrane, while the minority distributes in the cytoplasm in a punctuated manner. Transfected β-catenin localises at the cell membrane in the absence of HGF (C′), while it distributes in the cytoplasm in the presence of HGF (D′), similar to E-cadherin-positive MDCK cells (see Fig. 3J,K). Scale bar, 20 µm. (E) TOPflash reporter assay in MDCK cells (control, −Tet) or MDCK cells depleted of E-cadherin (E-cadherin shRNA, +Tet). Cells transfected with TOPflash reporter were either treated with Wnt3a medium or co-transfected with β-catenin. Treatment of cells with HGF enhances cells' response to Wnt3a or β-catenin, as seen in Fig. 1E and 1F. In cells depleted of E-cadherin, HGF fails to enhance the response to Wnt3a and β-catenin. (F) Western blot analysis of membrane (upper two rows) and cytosolic (lower four rows) fractions of MDCK cells without or with induction of E-cadherin shRNA expression, without or with HGF. Some groups of cells were treated with Wnt3a conditioned medium or with 2 µM BIO, a GSK3β inhibitor, or transfected with myc-tagged β-catenin, as indicated at the top. The membrane fraction analysis shows that E-cadherin shRNA completely abolishes E-cadherin protein. The cytosolic fraction was analysed with anti-Active-β-catenin (ABC) and anti-β-catenin (total) antibodies, along with anti-myc antibody to detect exogenous β-catenin and with anti-β-tubulin for loading control. Cell lysate of A431 human carcinoma was used as a positive control for anti-active-β-catenin antibody. In all experimental groups, active-β-catenin is increased in response to HGF when E-cadherin expression is kept intact. In contrast, in the absence of E-cadherin the ability of HGF to increase active-β-catenin is abolished. Note that myc-tagged β-catenin, larger than endogenous one in size, is also detected by ABC antibody, responding to HGF and to the loss of E-cadherin in a manner similar to endogenous ones treated with Wnt3a or BIO. (G) TOPflash reporter assay in MDCK cells (control, −Tet) or MDCK cells depleted of E-cadherin (E-cadherin shRNA, +Tet). Cells were transfected with TOPflash reporter along with β-catenin (β-cat) or the stabilised form in which four serine and threonine residues of GSK3β targets have been mutated to alanine (β4A) [45]. Similar to β-catenin, β4A construct does not show any significant activation of the reporter in E-cadherin-depleted cells, regardless of presence or absence of HGF.

As the above results suggest that the cadherin complex functions positively on β-catenin for activation of the Wnt pathway, we next examined Wnt pathway activation in the absence of E-cadherin. We used an MDCK cell line which stably carries an E-cadherin shRNA-expressing construct that is inducible by a Tet-ON system [40]. Depletion of E-cadherin was confirmed by Western blots (Fig. 5F). E-cadherin depletion in MDCK cells does not compromise cell adhesion or localisation of β-catenin at the cell membrane, presumably due to endogenous Cadherin-6 expression [41], although cells are slightly flattened (Fig. 5A,C). HGF causes further flattening of cells in the absence of E-cadherin (Fig. 5C,D). However, the majority of β-catenin remains at the cell membrane while the minority distributes in the cytoplasm in a punctated manner, different from the cells with E-cadherin (Fig. 5B,D). This suggests that intracellular translocation of β-catenin and its following distribution into the cytosol in response to HGF depends on internalisation with E-cadherin. A possibility remains that a depletion of E-cadherin might affect subcellular distribution of HGF receptor c-Met which is often associated with E-cadherin [42], [43] thus resulting in reduced HGF signalling. However, a complete blockage of HGF signalling is unlikely as cells do respond to HGF and show morphological changes (Fig. 5C,D).

In the TOPflash assay, HGF stimulation failed to activate the reporter in E-cadherin depleted cells (Fig. 5E). In addition, the response to both Wnt3a medium and β-catenin transfection is markedly impaired, suggesting a requirement for E-cadherin for β-catenin's transcriptional activity in MDCK cells. We further investigated whether E-cadherin knockdown affects the stability of cytosolic β-catenin by immunoblotting for the ‘active’ form of β-catenin in E-cadherin depleted cells following stimulation of the Wnt pathway by various methods such as treatment with Wnt3a or the GSK3β inhibitor BIO and transfection of *β-catenin* (Fig. 5F). The active form of β-catenin is detected by ABC antibody, which specifically recognises β-catenin that has not been phosphorylated by GSK3β at specific serine and threonine residues, and is hence not tagged for degradation [44]. Addition of Wnt3a or BIO in the medium causes an increase of active β-catenin, which is enhanced by addition of HGF (Fig. 5F). HGF also increases the amount of exogenous myc-tagged β-catenin that is found in the cytosol after transfection, and this corresponds with an increase in the immunoreactivity to ABC antibody, in agreement with the above TOPflash result (Fig. 5E,F). However, upon E-cadherin depletion, the ability of HGF to increase the active form of β-catenin is abolished in all conditions (Fig. 5F). In the immunocytochemical analysis, exogenously transfected β-catenin was found to be abundant in the cytoplasm in E-cadherin depleted cells in the presence of HGF, similar to parental MDCK cells treated with HGF (Fig. 5C′, D′), supporting the idea that availability of β-catenin in the cytoplasm does not reflect pathway activation. To further confirm the requirement for E-cadherin in β-catenin-dependent activation of the Wnt pathway, we used a mutated form of β-catenin that is resistant to degradation, and thus stable in the cytoplasm [45]. This stable form, in which four serine/threonine residues at the amino-terminal end are mutated to alanine (β4A), does not show any significant difference in TCF/LEF-dependent transcription compared to wild type β-catenin in MDCK cells in the absence of E-cadherin (Fig 5G), whereas the difference is noticeable in the presence of E-cadherin and HGF (Fig. 5G) and more pronounced in HEK293 cells (data not shown). However when E-cadherin is depleted, the stable form of β-catenin failed to activate the TOPflash reporter both in the absence and presence of HGF (Fig. 5G).

Collectively, these results show that E-cadherin is required for the augmented activation of the Wnt pathway in MDCK cells during HGF-dependent transformation. One might argue that attenuated TOPflash activity after E-cadherin depletion is due to failure of EMT, thereby keeping cells epithelial-like, the feature that renders cells less responsive to Wnt pathway activation (Fig. 1). The question, therefore, is what is the epithelial feature that prevents cells from activating the Wnt pathway? Our results of depletion of E-cadherin in the absence of HGF suggest that it is not E-cadherin that is responsible for preventing activation in epithelial cells (Fig. 5A,C,E), since depletion of it reduces activation, rather than enhancing it. Perhaps in MDCK cells, the cadherin complex serves as a pool of β-catenin, while exogenously overexpressed cadherins sequester β-catenin [9], [10], [11], [13], [14], [15]. Figure 5E together with Figure 3 clearly show that the effect of transfected β-catenin is most significant when E-cadherin is present and the cadherin complex undergoes internalisation. Therefore, the crucial events for β-catenin's activity seem to be firstly interacting with E-cadherin, either upon trafficking at ER/Golgi or transiently at the membrane, and second, becoming available in the cytoplasm. The latter step alone without a preceding interaction with cadherin does not provide transcriptionally active β-catenin even if it becomes abundant in the cytoplasm (Fig. 5D′). Our results also suggest that EMT in MDCK cells relies on an intact E-cadherin-based adhesion system and that HGF is unlikely to be able to induce cadherin-6 internalisation in a similar fashion to E-cadherin. These results highlight the fact that the ability of Wnt signals and exogenous β-catenin to activate transcription differs depending on the cellular context such as epithelial versus mesenchymal morphology and presence versus absence of E-cadherin. In other words, there are differences in the ability of cells to utilise β-catenin for transcription when it becomes available.

### Requirement for cadherins in activating the Wnt pathway *in vivo*

The above data suggest that cadherins are positive regulators of the Wnt pathway in two ways: by providing a pool of β-catenin and by rendering β-catenin competent in signalling. We wanted to examine whether this positive role is also true in *in vivo* settings. However, the fundamental role for E-cadherin in constructing and maintaining tissue structures impeded us to analyse the requirement of E-cadherin *in vivo*. In addition, although E-cadherin expression in MDCK cells is maintained during EMT, in *in vivo* contexts cells often switch the expression of cadherin types at the event of EMT. For example, in mouse 8.5 dpc embryos, epiblast cells forming the epithelial layer expresses E-cadherin, whereas the cells undergoing EMT in the primitive streak switch off E-cadherin and turn on N-cadherin [46]. These delaminated cells migrate laterally and differentiate into mesoderm. Notably, Wnt/β-catenin activity is known to be required for the ingressing cells to differentiate into mesoderm [19], [47], although ligand expression, such as of Wnt3a and Wnt8, is rather down-regulated upon delamination [19], [48]. We speculated that Wnt/β-catenin activity in cells undergoing EMT in this context is augmented by cadherin, particularly by N-cadherin which is expressed in cells becoming mesoderm during the whole process of ingression until they are re-epithelised to form somites [46], [49].

**Figure 6:**
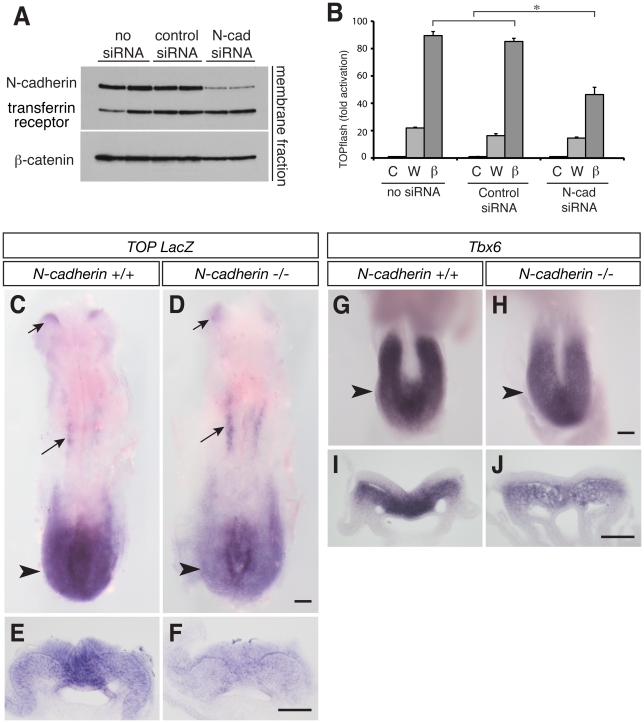
N-cadherin-deleted mouse embryos show low Wnt pathway activity and reduced downstream target gene expression during EMT. (A) Western blot analysis of membrane fraction of HEK293 cells transfected with control or N-cadherin siRNA. Transferrin receptor serves as a loading control. N-cadherin siRNA attenuates N-cadherin expression. β-catenin is also slightly reduced. (B) TOPflash reporter assay in HEK293 cells, after transfection of N-cadherin siRNA. Cells were either treated with control medium (C) or Wnt3a medium (W), or transfected with β-catenin (β). N-cadherin siRNA attenuates the effect of β-catenin transfection. *P<1×10^−6^. (C–F) TOP-LacZ reporter mouse embryos at 8.5 dpc with genotypes of wild type (+/+) or deleted (−/−) N-cadherin. The reporter expression is detected by an in situ hybridisation probe against LacZ. At the level of the primitive streak in the posterior region (arrowheads in C,D), the reporter expression is much weaker in the −/− mutant, while the midbrain (short arrows) and anterior somites (long arrows) show a comparable level of expression. (E) and (F) are transverse sections of (C) and (D), respectively, at the level of the primitive streak (arrowheads), where epiblast cells delaminate at the midline and migrate laterally to form mesoderm. (G–J) Mouse embryos at 8.25 dpc with wild type (G,I) or N-cadherin −/− (H,J) genotypes, stained with *Tbx6*, a Wnt signal target gene, by RNA in situ hybridisation. (G) and (H) are close-ups of the open neural plate region in the posterior. N-cadherin mutant embryos show a weaker Tbx6 expression. Transverse sections at the level indicated by arrowheads are shown in I and J. The expression level of *Tbx6* in delaminated mesodermal cells in the N-cadherin −/− embryo is significantly low compared to that of wild type. Scale bars, 100 µm.

As N-cadherin has distinct features from E-cadherin [50], [51], we first confirmed a positive role for N-cadherin in the transcriptional activity of β-catenin *in vitro*, using HEK293 cells, which strongly express N-cadherin (Fig. 6A,B). In the presence of N-cadherin siRNA, the transcriptional activity of β-catenin is significantly attenuated (Fig. 6A,B), supporting the requirement for cadherin in Wnt signalling.

We next analysed Wnt signalling readout in N-cadherin deleted mouse embryos [52], [53]. In the background of the TOP-LacZ reporter [54], N-cadherin-deleted mouse embryos show a significantly lower expression of the reporter in the posterior region, both in the delaminating cells at the midline of the epiblast and in laterally migrating mesodermal cells, while other regions such as the differentiating somites and the midbrain show comparable levels of reporter expression to that of N-cadherin +/+ embryos (n = 8 for each; Fig. 6C–F). In support of the reduced Wnt activity, the expression of *Tbx6*, a Wnt/β-catenin target gene required for mesodermal differentiation [18], [37], [47], [55], [56], was also attenuated in N-cadherin-deleted embryos (n = 4, Fig. 6G–J). Thus the positive regulation of the Wnt/β-catenin pathway by cadherin is not merely an *in vitro* phenomenon, but one that could affect signalling *in vivo*.

In summary, we have shown that (1) cadherins maintain a pool of β-catenin that can be made available for signal transduction upon EMT; (2) mesenchymally transformed cells are highly responsive to Wnt ligands and overexpressed β-catenin in the activation of the Wnt pathway; (3) the internalisation process, presumably that of the cadherin complex, is a prerequisite for the augmented activation upon EMT; and (4) cadherins are required for and play an instructive role in providing β-catenin for Wnt signalling, such that binding of β-catenin to cadherins renders β-catenin competent in the Wnt pathway.

## Discussion

In an effort to obtain *in vitro* data to explain the potential *in vivo* phenomenon of ligand-independent Wnt pathway activation, we have found that conversion of epithelial cells to a mesenchymal morphology activates the Wnt pathway in two ways. Firstly, as an immediate response, the conversion is associated with an increase of β-catenin in the cytosol, where it then becomes available to activate target genes, which we show to be dependent on endocytosis and independent of protein synthesis. Secondly, we show that the cellular changes that occur at EMT augment Wnt pathway readout by exogenous Wnt ligand and transfected β-catenin, and that this augmentation also requires intact endocytic processes. Moreover, we also find that cadherins play a positive role in β-catenin-dependent transcription: it is crucial for β-catenin to bind cadherin in order to activate downstream genes; β-catenin with reduced affinity for E-cadherin (the Y654E mutant) shows impaired activation of transcription, while loss of E-cadherin reduces Wnt transcriptional readout by both Wnt3a ligand and transfected β-catenin. Requirement of cadherins is further verified in an *in vivo* context, where knockout of N-cadherin leads to reduced Wnt pathway activity in ingressing cells during gastrulation.

### Mobilisation of β-catenin from cell membrane for use in signalling

We have shown that cells with distinct epithelial features are refractory to exogenous Wnt signals, in comparison to mesenchymal-like cells, but that their conversion to mesenchyme by HGF greatly facilitates the utilisation of β-catenin for signalling. This stimulation of canonical β-catenin signals by HGF, and the subsequent increase in transcriptional readout at the immediate-early stage, appears to be a result of movement of pre-existing β-catenin from the membrane to the cytoplasm, since it occurs independently of protein synthesis. This supports previous studies that suggest that β-catenin from the membrane can enter the nucleus and be used for transcription [8], [57], and puts forward a potential ligand-independent explanation for the lack of obligate correlation between Wnt ligand expression and Wnt pathway activation [16], [17], [19], [58]. Our results are also consistent with the observation that cell density affects the expression of E-cadherin as well as subcellular localisation of β-catenin [59], and that dissociation of cells in a low calcium condition *in vitro* causes an increase of cytosolic β-catenin in an ‘active’ form [60]. We find that the stimulation of β-catenin signals from the membrane is dependent on endocytosis, as treatment with the dynamin inhibitor dynasore attenuates augmentation of β-catenin-dependent transcription by HGF. We speculate that endocytosis in this EMT model is required at least for the turnover of E-cadherin and the dissociation of β-catenin from recently endocytosed E-cadherin, not only at the immediate-early stage but also when cells have become mesenchymal (Fig. 3). Interestingly, a study showed a requirement for endocytosis in the cadherin-free L cell line for stabilisation of β-catenin and activation of the TOPflash reporter in response to Wnt and lithium [61]. The exact role of endocytosis in the Wnt pathway has yet to be clarified, particularly with respect to the identity of what needs to be endocytosed, given the lack of cadherins in L cells, and how direct inhibition of GSK3β by lithium requires intact endocytosis processes.

### EMT facilitates activation of the Wnt pathway by Wnt signals

In addition to describing the mobilisation and transcriptional utilisation of β-catenin from the membrane, we demonstrate another fundamental cell biological phenomenon: that the cellular alterations occurring at EMT augment Wnt pathway readout in response to exogenous Wnt ligand and transfected β-catenin. The result that transfected β-catenin shows high transcriptional activity in cells that have undergone EMT, together with the results that both inhibition of internalisation and depletion of endogenous cadherin abolish the augmentation despite the presence of β-catenin in the cytoplasm, implies control at the subcellular, rather than extracellular, level. The increase in E-cadherin internalisation at EMT [21], [43] is one mechanism which might facilitate activation of the Wnt pathway, since it not only provides, but also augments, by increased turnover, a pool of signalling-competent β-catenin.

### Availability of β-catenin in the cytoplasm and pathway activation

It might be tempting to suggest that HGF potentiates transcriptional activity of β-catenin by increasing its stability. HGF does indeed increase the amount of stable β-catenin in the cytosol, as detected by ABC immunoblotting, and this is reflected in the augmented activity of TOPflash (Fig. 5). However, HGF also increases the total cytosolic β-catenin (Fig. 2,5), and hence we have yet to determine whether the absolute ratio of stable∶total β-catenin increases or whether HGF specifically increases the stability of β-catenin by preventing it from being phosphorylated. The destruction complex component APC is generally high in epithelial MDCK cells [62] and we did not detect any change in APC levels by HGF treatment (data not shown). In fact it has been reported that APC levels increase in HGF-treated cells relative to untreated cells [63]. It is also possible that HGF treatment caused a decrease in the level of GSK3β [64] or an increase in the levels of Ras or PI 3-kinase [65], [66], [67], all of which would increase the stability of cytosolic β-catenin. In our study, HGF augments transcriptional activity of exogenous β-catenin in both its native and stable forms, suggesting that this is a stability-independent event (Fig. 5G). In addition, the stable form of β-catenin is not active in MDCK cells in the absence of HGF (Fig. 5G). These results suggest that the role of HGF is not to specifically increase the stability of β-catenin. The findings that the effect of HGF is dependent upon endocytosis (Fig. 3) and E-cadherin (Fig. 5) also suggest that a direct stabilising function is unlikely. In support of the idea that stabilisation of β-catenin is not the sole determinant in its transcriptional activity, we show that in some experimental situations saturation of the cytoplasm with β-catenin is not reflected by an increase in transcriptional readout (Figs. 3I,L, 4). Similarly, it has been shown that increased cytosolic pools of β-catenin are not the cause of translocation into the nucleus in the case of melanoma cells [62]. These findings could have far-reaching implications, since it has always been considered that the key step in the Wnt pathway is regulation of the cytosolic pool of β-catenin that is available to enter the nucleus and thereby modulate transcription. One remaining possibility is that HGF potentiates transcriptional activation by inducing dissociation of β-catenin from α-catenin through ERK signalling [68]. However, our result that ERK phosphorylation is not affected by dynasore, while β-catenin's transcriptional activity is, suggests that the effect is ERK-independent.

### Requirement of cadherins in Wnt/β-catenin signalling

The result that cadherins are required for activation of the Wnt pathway, both in MDCK (Fig. 5) and HEK293 cells (Fig. 6B), was an unexpected one, given that cadherins are generally considered as negative regulators of Wnt pathway activation through sequestration of β-catenin. Indeed, forced overexpression of cadherins has been found to antagonise Wnt/β-catenin signalling in *Xenopus*, *Drosophila* and cell culture models [11], [13], [14], [69], [70]. Loss of E-cadherin has been reported to induce colocalisation of β-catenin with LEF1 in the nucleus [13], whilst it has been suggested that the downregulation of E-cadherin during gastrulation results in a release of membrane-bound β-catenin, allowing for its nuclear translocation and an increase in Wnt signalling [71]. This has led to the hypothesis that the level of cadherin expression in a cell sets a threshold over which β-catenin must accumulate in order to translocate into the nucleus and drive transcription. However, as aforementioned, we find that there is no correlation between cytoplasmic accumulation of β-catenin and transcriptional readout.

Importantly, however, it has been shown more recently that whilst knockdown of E-cadherin augments β-catenin-dependent transcription in colon cancer cells in which the Wnt pathway is active, it has no effect in nontransformed keratinocytes that do not display Wnt signalling [72]. Similarly, cancer cell lines that lack E-cadherin do not exhibit a corresponding upregulation of β-catenin signalling [73], [74]. Moreover, depletion of E-cadherin in a mouse model for pancreatic cancer (the Rip1Tag2 transgenic mouse) was found not to contribute to Wnt/β-catenin signalling [75]. These data indicate that the mere loss of E-cadherin is not sufficient to activate β-catenin signalling. Although, in cells in which the degradation machinery is compromised, or in cells already actively engaged in Wnt signalling, loss of cadherin might act to amplify the response to Wnt signals. For example, introduction of β-catenin containing the Y654E mutation increases TCF-mediated transcription [34], [76]. This is opposite to our result that the Y654E mutant is less competent to activate transcription (Fig. 5). However, it is important to note that the aforementioned studies used either mouse embryonic fibroblasts [76] or SW-480 cells [34], which contain very little E-cadherin or/and have a compromised β-catenin degradation pathway due to truncation of APC [77], [78].

Our data confirm that cadherin loss does not activate β-catenin signalling, and suggest that the endocytosis of – rather than the transcriptional downregulation of – cadherins can promote Wnt signalling. We would argue that earlier suggestions that cadherin levels set a threshold for Wnt/β-catenin signalling are only partly correct. In MDCK cells, we propose that the high levels of E-cadherin on the cell membrane keep the Wnt pathway off, and that application of Wnt3a ligand or transfection of β-catenin has no significant effect on pathway readout due to the strength of the E-cadherin/β-catenin interaction. Pretreatment with HGF greatly increases readout by both Wnt and β-catenin. This is not due to the downregulation of E-cadherin, but probably due to increased endocytosis and turnover of E-cadherin by HGF, which lead to increased β-catenin being released into the cytoplasm. In fact, E-cadherin levels in the membrane fraction increase with HGF treatment (Figs 2G, 4F). This increase might be a byproduct of increased turnover and trafficking to the membrane and may account for HGF's ability to potentiate the Wnt response. Furthermore, although very high levels of cadherin might inhibit Wnt pathway activity, too little also does; knockdown or loss of cadherin results in a significantly reduced activation by β-catenin and Wnt in both *in vitro* and *in vivo* contexts (Figs 5, 6). Moreover, β-catenin that has reduced affinity for E-cadherin (Y654E) is far less able to function in transcription (Fig. 4). So it seems that binding of β-catenin to E-cadherin is required for its proper function, but that too much E-cadherin can negatively affect the pathway by sequestration.

### The positive role of cadherins in Wnt/β-catenin signalling

How might cadherin function to positively regulate the Wnt pathway? It is obvious to think that cadherins could promote Wnt signalling by stabilising β-catenin (albeit at the membrane) by outcompeting β-catenin's alternative binding partner, the Axin/APC degradation complex [75], [79]. Thus with cadherin loss, more β-catenin becomes available for consumption by the degradation complex. This supports the idea that cadherin loss could not increase β-catenin stabilisation unless the degradation machinery was somehow compromised [72]. However, we find that β-catenin stabilisation and/or accumulation in the cytoplasm does not always correlate with transcriptional activation. We postulate that cadherins play a more instructive role in Wnt signalling; that binding to and subsequently dissociating from cadherin somehow ‘primes’ β-catenin for utilisation in signalling. We do not know what this priming event involves, but, given the reduced transcriptional activation by Y654E, do not believe it to be phosphorylation at this tyrosine residue. It has previously been shown that Wnt signalling causes β-catenin to preferentially bind TCF over cadherin [2]. It is interesting to propose that binding to cadherin might generate a molecular form of β-catenin that, once dissociated, is more active in signalling and that the effect on signalling that differential binding affinities has is different in different cell types, depending on the biological needs of the cells/tissues and the identity of the ligands to which they are responding. For example, in the absence of Wnt ligands but presence of signals capable of inducing EMT, transcriptional activation by β-catenin can still occur through utilisation of ‘primed’ β-catenin from the membrane. In the presence of Wnt ligands, however, β-catenin binds TCF over cadherin [2], thereby bypassing the need for this priming event. Perhaps this goes some way in explaining why N-cadherin knockdown in HEK293 cells reduces readout by β-catenin, but not by Wnt3a ligand (Fig. 6B); treatment with Wnt3a generates a TCF-selective β-catenin that is not affected by N-cadherin knockdown, but in order for β-catenin, introduced directly into the cell by transfection, to be utilised for signalling, it must first bind to and be primed by N-cadherin.

It has been shown that newly synthesised β-catenin is bound to E-cadherin in the ER and that this coupling facilitates efficient exit of the complex from the ER and its subsequent trafficking to the basolateral membrane [80], [81]. One possible difference between overexpressed β-catenin and endogenous β-catenin is a lack of this interaction, due to shortage of the corresponding amount of endogenous E-cadherin. Overexpressed β-catenin may be released from the ER/Golgi into the cytoplasm without this coupling process, and then accumulate in the cytoplasm, as seen in Fig. 3K. It appears that cells with cadherin expression, active cadherin turnover and intact endocytosis processes, together with a mesenchymal morphology, are able to utilise overexpressed β-catenin for signalling. It is not clear why MDCK cells treated with HGF treatment are able to retain exogenous β-catenin in the cytoplasm while untreated cells that maintain an epithelial morphology are not (Fig. 3J,K). Perhaps differential β-catenin retention is an intrinsic property of cells influenced by the events at EMT and/or cell morphology.

### Interplay between cell shape changes and pathway activation

There are a number of examples where cell shape affects pathway activation and, in turn, gene regulation. For example, mesenchymal stem cells differentiate into either adipocytes or osteoblasts *in vitro*, with the lineage decision depending on the surface area of the substrate on which the cells are allowed to grow [82]. In *Drosophila* embryos, mechanical deformation during morphogenetic movement induces specific gene expression [83]. It is worth noting that both cases involve regulation of Wnt/β-catenin signalling [83], [84], [85]. Perhaps sharing β-catenin between the adhesion system and signalling is a method cells have adopted in order to reflect morphological changes in gene regulation.

## Materials and Methods

### Cell culture

HEK293T (ATCC) and MDCK cells [40] were maintained in DMEM with 10% FCS and penicillin/streptomycin. E-cadherin shRNA MDCK cells [40] were maintained additionally with 800 µg/ml G418 and 5 µg/ml blasticidin and the expression of shRNA was induced by adding 2 µg/ml tetracycline 48 hours before use. Wnt3a conditioned medium was collected from L cells stably expressing Wnt3a [86] and diluted 1∶1 with the growing medium. Human recombinant HGF (R&D) was used at 250 ng/ml and cycloheximide at 20 µg/ml. HGF was applied in the last 16 hours before collection unless stated otherwise. When using cycloheximide and Wnt3a, cells were first treated with cycloheximide alone for 30 minutes, followed by a treatment with a mix of fresh cycloheximide and Wnt3a for 4 hours. Similarly, cells were treated with 50–400 µM Dynasore (Sigma) one hour before adding HGF.

### Transfection and reporter assays

TOPflash reporter (Upstate) and Renilla luciferase (Promega), together with control or β-catenin DNA where necessary, were transfected using Polyfect (Qiagen) following the manufacturer's protocol. Cells were lysed 2 days after transfection and processed for the Dual Luciferase Assay System (Promega). All data were triplicate and analysed using student's t-test. For N-cadherin siRNA, lipofectamine 2000 (Invitrogen) was used following the manufacturer's protocol. Control and N-cadherin siRNA were obtained from Santa Cruz (sc-44232, sc-29403, respectively).

### Protein analyses

To collect cytosolic and membrane fractions, cells were grown in 35 mm dishes and were collected with 100 µl cold hypotonic buffer (10 mM TrisHCl pH 7.5, 0.2 mM MgCl_2_, with proteinase inhibitor cocktail (Roche) and 1 mM AEBSF) with a cell scraper. The extract was dounce homogenised and spun at 15,000 g for 45 minutes at 4°C, and the supernatant was collected as the cytosolic fraction. The pellet was resuspended in 100 µl lysis buffer (150 mM NaCl, 20 mM TrisHCl pH 7.5, 1% Triton X-100, proteinase inhibitor cocktail (Roche) and 1 mM AEBSF). After keeping on ice for 10 minutes, it was spun at 10,000 g for 10 minutes at 4°C, and the supernatant were collected as the membrane fraction. Both fractions were incubated with Laemmli sample buffer containing 5% β-mercaptoethanol at 100°C for 5 minutes before loading onto the gel. Antibodies used for Westerns are; anti-β-catenin (Sigma), anti-active-β-catenin (anti-ABC, clone 8E7, Millipore), anti-E-cadherin (BD Bioscience), anti-β-tubulin (Sigma), anti-transferrin receptor (Santa Cruz), anti-flag (Sigma) and anti-myc (Sigma). A431 cell lysate as a positive control for anti-active-β-catenin antibody was provided by Millipore.

### Immunocytochemistry

Cells grown on coverslips were fixed with 3% paraformaldehyde in PBS for 30 minutes at 4°C, followed by incubation in 50 mM NH_4_Cl and 0.2% Saponin in PBS for 15 minutes at room temperature. After washing with PBS containing 0.2% gelatin and 0.02% Saponin, cells were incubated with primary and then secondary antibodies. Antibodies used are; anti-β-catenin (Abcam), anti-E-cadherin (BD Biosciences), anti-transferrin receptor (Invitrogen), anti-Flag (Santa Cruz).

### Q-PCR analyses

MDCK cells were transfected with TOPflash reporter DNA. On the next day, cells were split into small dishes and treated with HGF and/or cycloheximide for 6 hours. RNA was collected using Trizol (Invitrogen) following manufacturer's protocol. Additional DNase treatment was performed to eliminate any contamination of DNA. After cDNA synthesis, PCR reaction was performed using Express SYBR GreenER (Invitrogen) on ABI 7500 (Applied Biosystems). Data of triplicate average Ct were normalised with that of GAPDH and then further converted to fold difference relative to no-HGF, following manufacture's protocol (Applied Biosystems). P-values were calculated using ΔC_T_ against GAPDH using student's t-test.

### Mouse mutant analyses

N-cadherin flox mice with LoxP sites at the regions flanking the exon 1 of N-cadherin [52] were mated with mice carrying the Cre transgene expressed under a β-actin promoter [87]. Genotyping was performed as described [52]. Individual embryos were fixed with 4% paraformaldehyde in PBS and processed for in situ hybridisation with LacZ or Tbx6 probes.
